# Measurement of Redox Biomarkers in the Whole Blood and Red Blood Cell Lysates of Dogs

**DOI:** 10.3390/antiox11020424

**Published:** 2022-02-19

**Authors:** Luis G. González-Arostegui, Alberto Muñoz-Prieto, Asta Tvarijonaviciute, José Joaquín Cerón, Camila Peres Rubio

**Affiliations:** 1 Interlab-UMU, Regional Campus of International Excellence “Mare Nostrum”, University of Murcia, 30100 Murcia, Spain; luisgarostegui@gmail.com (L.G.G.-A.); alberto.munoz@um.es (A.M.-P.); asta@um.es (A.T.); jjceron@um.es (J.J.C.); 2 Clinic for Internal Diseases, Faculty of Veterinary Medicine, University of Zagreb, Heinzelova 55, 1000 Zagreb, Croatia; 3 Department of Animal and Food Science, School of Veterinary Science, Universitat Autònoma de Barcelona, 08193 Cerdanyola del Vallès, Spain

**Keywords:** antioxidants, biomarkers, dogs, oxidants, whole blood

## Abstract

The evaluation of the biomarkers of oxidative status is usually performed in serum, however, other samples, such as red blood cells (RBCs) lysates or whole blood (WB), can be used. The objective of this study was to evaluate if a comprehensive panel of redox biomarkers can be measured in the WB and RBCs of dogs, and their possible changes “in vitro” after the addition of different concentrations of ascorbic acid. The panel was integrated by biomarkers of the antioxidant status, such as cupric reducing antioxidant capacity (CUPRAC), ferric reducing ability of plasma (FRAP), Trolox equivalent antioxidant capacity (TEAC), thiol and paraoxonase type 1 (PON-1), and of the oxidant status, such as total oxidant status (TOS), peroxide-activity (POX-Act), reactive oxygen-derived compounds (d-ROMs), advanced oxidation protein products (AOPP) and thiobarbituric acid reactive substances (TBARS). All the assays were precise and accurate in WB and RBCs lysates. In addition, they showed changes after ascorbic acid addition that are in line with previously published results, being WB more sensitive to detect these changes in our experimental conditions. In conclusion, the panel of assays used in this study can be measured in the WB and RBCs of the dog. In particular, the higher sensitivity to detect changes in our experimental conditions and its easier sample preparation makes WB a promising sample for the evaluation of redox status in dogs, with also potential applications to other animal species and humans.

## 1. Introduction

The oxidation–reduction (redox) homeostasis is of high importance to life, being involved in the most important biological processes [1]. Redox status can be assessed by the measurement of oxidant substances such as reactive oxygen species (ROS) [2,3] and by the evaluation of the antioxidant status, using biomarkers of the total oxidant capacity (TAC) as the cupric reducing antioxidant capacity (CUPRAC), ferric reducing ability of plasma (FRAP) and Trolox equivalent antioxidant capacity (TEAC), as well as, individual antioxidant compounds such as thiol, superoxide dismutase (SOD), glutathione peroxidase (GPx) and paraoxonase type-1 (PON-1) [4,5,6,7]. Oxidative stress is defined as a disturbance in the redox balance directed towards oxidation, which can produce cellular and tissue damage [8].

The evaluation of biomarkers of redox status is usually performed in serum, nonetheless, other samples, such as red blood cells (RBCs) lysates, whole blood (WB), saliva and urine, can be used. The involvement of RBCs in the measurement of redox analytes have the potential advantage of being more sensitive to oxidative stress due to their role in oxygen transport, making them one of the first blood components affected by oxidative stress [9,10]. In addition, WB serves as a reflection of the overall redox balance of other tissues [11], making it an interesting sample type to study antioxidants and oxidants [12].

In dogs, the use of RBCs lysates and WB has been proven to be effective in the measurement of selected antioxidants and oxidants. In RBCs lysates, SOD was measured to evaluate the antioxidant response of dogs receiving a diet consisting of high polyunsaturated fatty acids [13], as well as in dogs with chronic kidney disease [14], atopic dermatitis [15], parvoviral infection [16], sarcoptic mange [17], leishmaniasis [18] and uncomplicated babesiosis [19]. In addition, reduced glutathione has been measured in RBCs lysates from dogs with renal azotemia [20] and GPx has been determined in RBCs lysates from dogs infected with *Dirofilaria immitis* [21] and with multicentric lymphoma [22]. Among oxidants, thiobarbituric acid reactive substances (TBARS) have been used to evaluate lipid peroxidation in the RBCs lysates of dogs with a diet consisting of high polyunsaturated fatty acids [13], as well as in dogs with sarcoptic mange [17] and dirofilariasis [21]. Regarding WB, GPx has been measured in a storage study [23], as well as in dogs with leishmaniasis [18] and heart failure [24].

In vitro studies in which ascorbic acid (vitamin C) is added to the sample have been performed previously as a model to evaluate the ability of different analytes to detect changes in the redox status of the sample [25]. Ascorbic acid is a known chain-breaking antioxidant that can also act as a pro-oxidant under some specific conditions in biological systems [26].

To the author’s knowledge, the assessment of oxidative stress in dogs using samples such as WB and RBCs has been limited to the evaluation of SOD, reduced glutathione, GPx activity and TBARS in RBCs and GPx activity in WB, as previously indicated. Therefore, the objective of this study was to evaluate if a comprehensive panel of redox biomarkers, integrated by 10 analytes that are commonly measured in canine serum, could also be measured in WB and RBCs, and if they could show changes after an “in vitro” addition of an antioxidant. For this purpose, in this report, the following activities were performed: (1) an analytical validation in WB and RBCs lysates of biomarkers of the antioxidant status, such as CUPRAC, FRAP, TEAC, thiol and PON-1, and of the oxidant status, such as total oxidant status (TOS), peroxide-activity (POX-Act), reactive oxygen-derived compounds (d-ROMs), advanced oxidation protein products (AOPP) and TBARS; and (2) an evaluation of these biomarkers after the addition of an antioxidant compound, such as ascorbic acid, at two different concentrations, situations that are known to produce changes in the redox status of the sample.

## 2. Materials and Methods

### 2.1. Sample Preparation

Blood samples were obtained from clinically normal Beagle dogs that belonged to the Animal Resources Center of the University of Murcia. All samples were collected by jugular venipuncture and placed in EDTA tubes and analyzed for a complete blood count. Each blood sample was then divided into two different aliquots for posterior WB and RBCs preparation and plasma separation. To obtain the WB, one aliquot was kept at −80 °C for at least two hours before the analysis of the oxidative status biomarkers. The other one was used to prepare the RBCs lysate by osmotic shock, as reported previously [25,27,28]. For this purpose, the sample was centrifuged at 3000 rpm × 10 min at 4 °C. Subsequently, plasma was separated and stored in Eppendorf tubes, the buffy coat was discarded, and the RBCs were washed with isotonic saline (NaCl 0.9%) and centrifuged, as previously mentioned. Then, the supernatant was removed, and the process was repeated. After a total of four washes, the packed RBCs were hemolyzed in a 1:4 dilution using ultrapure water and then stored at −80 °C until analysis.

### 2.2. Assays

#### 2.2.1. Antioxidant Status

CUPRAC assay was based on the reduction of Cu^2+^ into Cu^1+^ by the nonenzymatic antioxidants in the sample [29]. Evaluation of CUPRAC was made following the procedure previously validated for use in serum of dogs [30]. Results are expressed in millimoles per liter of sample (mmol/L).

FRAP assay was based on the reduction of ferric-tripyridyltriazine (Fe^3+^-TPTZ) to the ferrous (Fe^2+^) form [31]. Its determination was made following previously described methods [31,32]. Results are expressed in mmol/L. 

Measurement of TEAC was based on the assay described by Arnao et al. [33], which has been used previously in canine serum samples [34]. Its principle is based on the enzymatic generation of 2,2′-azino-bis(3-ethylbenz-thiazoline-6-sulfonic acid) (ABTS) radical and its reduction by nonenzymatic antioxidants presents in the sample [33]. Results are expressed in mmol/L.

The determination of total thiol is based on the reaction of thiols within the sample with 5,5′-dithiobis-(2-nitrobenzoic acid) (DTNB). The assay used was performed according to previously described methods for serum samples [35,36]. Results are expressed in micromoles per liter (µmol/L). 

Measurement of PON-1 was based on the hydrolysis of phenylacetate into phenol and it was determined as previously described in canine serum [37]. Results are expressed in international units per milliliter of sample (IU/mL).

#### 2.2.2. Oxidant Status

The determination of TOS was based on the assay described by Erel [38], which was previously used in dogs [39]. Its reaction is based on the ability of oxidants in the sample to oxidize Fe^2+^-*o*-dianisidine complex to Fe^3+^ [38]. Results are expressed in µmol/L.

The POX-Act assay was based on the determination of total peroxides through a peroxide–peroxidase reaction using tetramethylbenzidine as the chromogenic substrate [40]. Determination of POX-Act was measured following a validated method for human sera [40]. Results are expressed in µmol/L.

The determination of d-ROMs assay was based on the reaction of the sample in an acidic medium in the presence of *N*,*N*,-diethyl-*para*-phenylenediamine (DEPPD), and it was made following a previously described method [41]. Results are expressed in Carratelli Units (U.CARR).

AOPP determination was based on oxidized albumin and di-tyrosine containing cross-linked proteins, as previously described [42] and measured in canine serum [43]. Results are expressed in µmol/L.

Determination of TBARS is based on the reaction of the sample to a trichloroacetic acid, thiobarbituric acid and *N* hydrochloric acid stock (15% *w*/*v* trichloroacetic acid; 0.375% *w*/*v* thiobarbituric acid; 0.25 *N* hydrochloric acid) in heated conditions [44]. TBARS was measured following a previously described method [44] using a microplate reader (Powerwave XS, Biotek Instruments, Winusky, VT, USA). Results are expressed in µmol/L.

### 2.3. Analytical Validation

Analytical performances of the assays were assessed by calculating precision, accuracy and detection limit according to the protocol previously described by Tiwari and Tiwari [45] that has been applied in other analytical validations in dogs [30,34,37].

#### 2.3.1. Precision

Precision was expressed as the coefficient of variation (CV; mean divided by standard deviation [SD] and multiplied by 100) and was calculated as inter- and intra-assay variation. Intra-assay precision was determined as the CV between five replicates from two WB and RBCs lysates samples (one with high and one with low concentration) in a single assay run. Inter-assay precision was determined as the CV between five replicates from two WB and RBCs lysates samples (one with high and one with low concentration) measured on five separate days. Samples used for the evaluation of the intra-assay CV were aliquoted in five different Eppendorf tubes and used for the determination of the inter-assay CV. Each aliquot was measured on a different day.

#### 2.3.2. Accuracy and Limit of Detection

Accuracy was evaluated through linearity under dilution and spiking recovery. To study linearity under dilution, the canine RBCs lysate samples were diluted with a Phosphate buffer pH 7.5 at 1:2, 1:4, 1:8, 1:16 and 1:32, and WB samples were diluted at the same dilutions and 1:25, 1:50, 1:100, 1:200 and 1:400. In order to study spike recovery, one sample containing high concentrations of each analyte and one sample containing low concentrations of each analyte were selected and mixed at different percentages (87.5%, 75%, 50%, 25% and 12.5% of the sample with high concentration with 87.5%, 75%, 50%, 25% and 12.5% of the sample with low concentration of each method, respectively). The ratios of the measured values to the expected values of each method were then calculated.

The limit of detection (LOD) for each assay was evaluated based on the data from 20 replicate determinations of phosphate buffer pH 7.5.

### 2.4. “In Vitro” Test

In brief, 5 mL of blood samples were drawn from the jugular vein of 7 male adult Beagle dogs (aged between 4–9 years) and collected in EDTA tubes. Each sample was divided into three groups: (1) control; (2) ascorbic acid 10 mM (VC10); and (3) ascorbic acid 60 mM (VC60). All plastic vials of all groups contained 940 µL of blood. To the control group vials, 60 µL of NaCl 0.9% were added. To the VC10 group vials, 60 µL of a 166.67 mM ascorbic acid solution were added. In the case of the VC60 group vials, 60 µL of 1 M ascorbic acid solution were added. The content of the vials was mixed and incubated for 2 h at 4 °C. After that, the WB, RBCs lysates and plasma samples were prepared as described in “sample preparation”. For the analysis, the WB and RBCs lysates were diluted with a Phosphate buffer pH 7.5 to achieve a concentration that allows the measurement of each analyte. Final concentrations of both WB and RBCs lysates were corrected by the dilution factor which varied depending on the analyte measured (ranging from 1:10 to 1:30) and were expressed in concentration or activity per volume of sample.

The procedures were approved by the University of Murcia’s ethics committees and the Ministry of Agriculture, Livestock, Fishing, and Aquaculture of the Region of Murcia (A13170503).

### 2.5. Statistical Analysis

Data were analyzed using GraphPad Prism software (GraphPad Software Inc., version 9.3 for MacOS). Arithmetic means, medians, intra- and inter-assay CVs were calculated by use of routine descriptive statistical procedures and computer software (Excel 2020, Microsoft; GraphPad Statistics Guide). Linearity under dilution was investigated by linear regression. The Shapiro-Wilk test was first used to assess whether data of the “in vitro” test were normally distributed. Differences in the concentrations between groups, when data were normally distributed, were assessed using a 2-way ANOVA followed by Tukey’s range test, and non-normally distributed datasets were assessed employing a 2-way ANOVA followed by Kruskal–Wallis test. Statistical differences were considered for *p*-values < 0.05.

## 3. Results

### 3.1. Analytical Validation

For WB samples, all assays showed an intra-assay CV between 0.01% and 15.89% and an inter-assay CV between 0.01% and 13.62% (Table S1). Serial dilution of WB resulted in linear regression higher than 0.9667 (Figure S1). Recovery in all cases was between 92.77% and 122.49%. 

For RBCs lysates, all assays showed an intra-assay CV between 1.02% and 15.98% (Table S2) and an inter-assay CV between 0.58% and 12.6%. Serial dilution of RBCs lysates resulted in linear regression higher than 0.96 for all assays studied (Figure S2). Recovery in all cases was between 87.5% and 113.9%.

The limit of detection for CUPRAC, FRAP, thiol, PON-1, TOS, POX-Act, d-ROMs, AOPP and TBARS was of 0.021 mmol/L, 0.013 mmol/L, 47.67 µmol/L, 0.12 IU/mL, 11.92 µmol/L, 5.28 µmol/L, 20.31 U.CARR, 16.16 µmol/L and 1.06 µmol/L, respectively. In the case of TEAC, values obtained were all negative and the limit of detection could not be calculated. 

### 3.2. “In Vitro” Test

#### 3.2.1. Antioxidant Status

**Figure 1:**
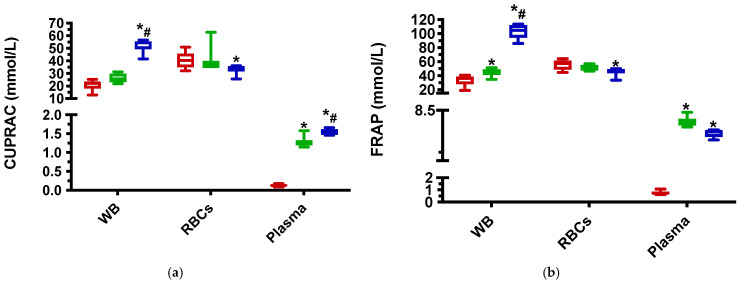

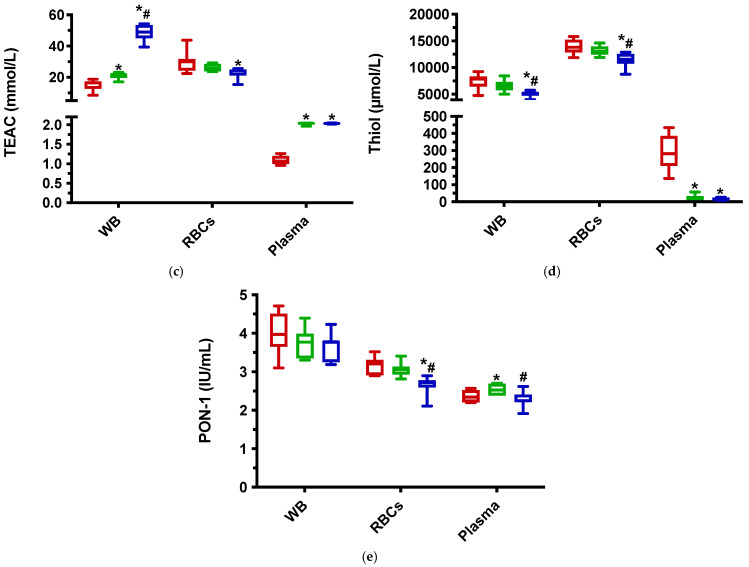
Results of the “in vitro” test on antioxidant biomarkers. (**a**) Cupric reducing antioxidant capacity (CUPRAC); (**b**) ferric reducing ability of plasma (FRAP); (**c**)Trolox equivalent antioxidant capacity (TEAC); (**d**) thiol, and (**e**) paraoxonase type 1 (PON-1) results obtained during the “in vitro” study for whole blood (WB), red blood cells (RBCs) lysates and plasma samples. Probability levels of *p* < 0.05 were regarded as significant and marked with an asterisk (*: vs. control) and a hashtag (#: VC10 vs. VC60). Red box: control group; green box: VC10 group; and blue box: VC60 group.

Results for antioxidant biomarkers in the “in vitro” test are shown in Figure 1. After the addition of ascorbic acid at 10 mM, the three TAC assays FRAP, TEAC and CUPRAC, showed significantly higher levels than the control group when measured in WB and plasma (except for CUPRAC in WB). In RBCs lysates, no significant differences were found in any assay. In plasma, the ascorbic acid at 10 mM produced a significant decrease in thiol. 

With ascorbic acid at 60 mM, in WB the three TAC assays showed significantly higher levels than control, and the magnitude of increases was significantly higher than with 10 mM. The TAC assays also showed significantly increased concentrations in plasma. However, in RBCs lysates, the three TAC assays were significantly lower than in the control group. Thiol was significantly lower than control in the three types of samples. PON1 was lower than control and VC10 in RBCs lysate. 

#### 3.2.2. Oxidant Status

**Figure 2:**
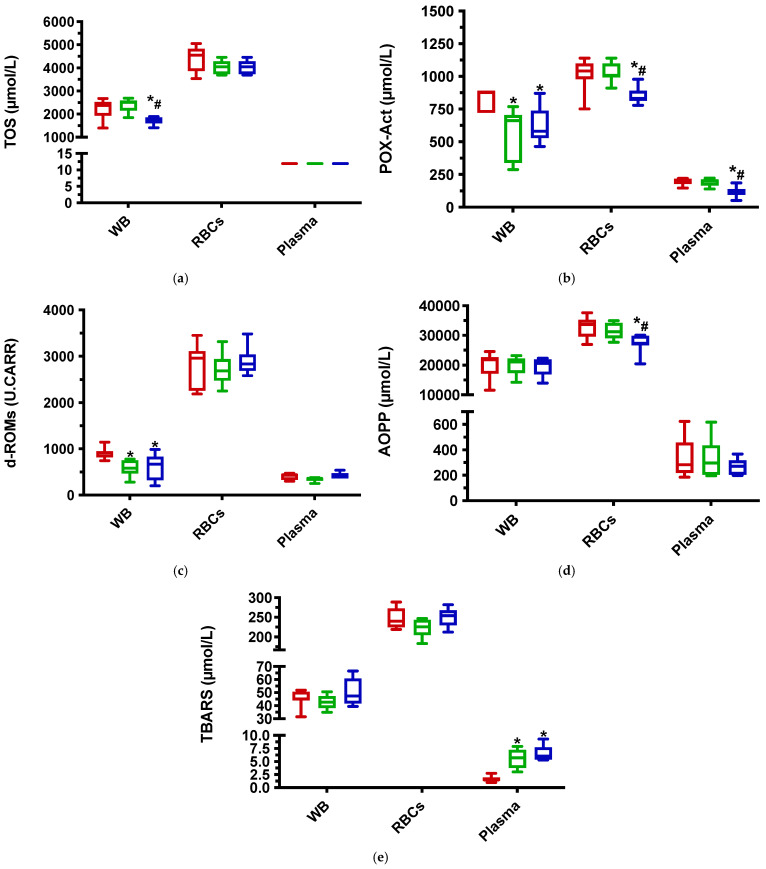
Results for the “in vitro” test on oxidant biomarkers. (**a**) Total oxidant status (TOS); (**b**) peroxide-activity (POX-Act); (**c**) reactive oxygen-derived compounds (d-ROMs); (**d**) advanced oxidation protein products (AOPP), and (**e**) thiobarbituric acid reactive substances (TBARS) results obtained during the “in vitro” study for whole blood (WB), red blood cells (RBCs) lysates and plasma samples. Probability levels of *p* < 0.05 were regarded as significant and marked with an asterisk (*: vs. control) and a hashtag (#: VC10 vs. VC60). Red box: control group; green box: VC10 group; and blue box: VC60 group.

Results for antioxidant biomarkers in the “in vitro” test are shown in Figure 2. After the addition of ascorbic acid at 10 mM, POX-Act and d-ROMs were significantly lower than control in WB. No significant changes were found in RBCs. In plasma, TBARS was higher than control.

With ascorbic acid at 60 mM, POX-Act and d-ROMs, and in addition, TOS were significantly lower than control in WB. In RBCs lysate, d-ROMs values were not different to control, but POX-Act and AOPP were significantly lower than control and VC10. In plasma, POX-Act was significantly lower and TBARS was significantly higher than control.

## 4. Discussion

In this study, a panel of assays that can be used for the evaluation of oxidative stress was validated in WB and RBCs lysate samples from dogs. Some of the validated analytes, to our knowledge, have not been previously studied in these types of samples. Therefore, to the author’s knowledge, this is the first study to report the use of CUPRAC, FRAP, TEAC, thiol, PON-1, TOS, d-ROMs, POX-Act and AOPP in WB and RBCs lysate samples from dogs.

The results of the analytical validation showed that all methods included in this study demonstrated adequate intra- and inter-assay CVs, as well as good linearity under dilution [45], similar to results obtained previously in serum of dogs [30,32,34,37]. In addition, all methods showed a high recovery. This would indicate that the assays validated in this study are precise and accurate and could be used to evaluate biomarkers of oxidative stress in WB and RBCs lysate samples from dogs. The use of automated assays leads to faster results and reduces error range. All the assays in this study were validated using an automatic biochemistry analyzer, except for TBARS. Nonetheless, the assays that were used through an automated analyzer can also be adapted to microplate readers and manual spectrophotometers.

As a second part of the study, the assays were used for the measurement of analytes in WB and RBCs in a situation that can change the oxidative status of the sample, as the addition of ascorbic acid. In this experiment, the analytes were also measured in plasma for comparative purposes. Ascorbic acid acts as an antioxidant since it participates in the reduction of O_2_^•−^ and lipid peroxyl radicals in both RBCs membranes and plasma [46,47,48,49] and it can inhibit the damage of RBCs [50]. However, in some specific conditions, ascorbic acid can lead to the production of H_2_O_2_ via reactions with metals such as Cu^2+^ and Fe^2+^ as well as with oxygen or by its self-oxidation [26].

The fact that TACs assays such as CUPRAC, FRAP and TEAC measure ascorbic acid could be the reason why these assays showed increased values in WB when ascorbic acid was added, with the increase being in a dose-dependent manner. However, this event was not evident when TACs were measured in RBCs samples, in which even a decrease was observed when 60 mM of ascorbic was added. This is in line with previous findings reporting lower CUPRAC values in erythrocytes at high concentrations of ascorbic acid [25]. We hypothesize that during the sample processing the compounds present in lysate samples, especially those released from lysed cells such as Fe, interact with the high concentrations of ascorbic acid, leading to a degradation of antioxidants present in the RBCs lysate sample. On the other hand, the concentrations of PON-1 in RBCs lysates and plasma and thiol in all sample types decreased when incubated with higher concentrations of ascorbic acid. It has been demonstrated that acid ascorbic can oxidize thiols by its auto-oxidation to dehydroascorbic acid, and, by consequence, the decreased PON1 activity due to the reduction in its free SH groups number could explain the findings of this study [51,52]. 

Regarding the oxidant status, it was found that the addition of 60 mM of ascorbic acid led to lower levels of POX-Act in all three types of samples, being indicative of the inhibition of peroxides formation by the added antioxidant. The concentrations of TOS and d-ROMs were also reduced in high ascorbic acid concentrations, but only when measured in WB. Overall, this would indicate that ascorbic acid, in the concentrations used, can inhibit oxidant compounds in biological samples, with POX-Act being more sensitive in detecting this effect. The increase in the oxidant compounds due to the liberation from lysed cells could be the cause of the lack of reduction of these markers in RBCs lysates, therefore RBCs lysates would be less sensitive than WB to detect the changes produced by ascorbic acid. 

During the experimental conditions, the use of WB was easier when compared with the RBCs lysates and plasma/serum, which are the samples traditionally used. For the obtention of plasma or serum, centrifugation and aspiration are needed. In addition, the preparation of lysate takes around one or two hours, depending on the chosen procedure [16,53]. In contrast, the preparation of WB samples is far more simple, since no centrifugation and washing procedures are needed. In addition, the higher concentration of most analytes in WB compared to plasma, possibly due to the intracellular antioxidants present in RBC and other blood cells, and the antioxidants bound to their surfaces [12].would be in favor of using this type of sample. Also, the “in vitro” study indicated that WB reflected better the expected changes after ascorbic acid addition than RBCs lysates or plasma. However, overall, this work should be considered as a pilot study since only an “in vitro” test was performed, and further studies involving larger populations of dogs should be performed to evaluate the changes in these analytes in the different type samples in physiological conditions and different diseases. As a part of these studies, a comparison of the analytes between serum and plasma would be of interest. The results of these studies will determine which type of sample is more adequate for the measurement of the different redox analytes in the dog. In addition, in our conditions, the hemoglobin concentration of the samples did not affect the results obtained in this work, therefore no corrections were made, and the values were only expressed in concentration or activity of the analyte per volume of the original sample. However, it would be interesting to confirm these findings in disease conditions in which alterations of hemoglobin and/or RBCs concentrations would occur. 

## 5. Conclusions

A panel of assays for evaluation of the redox status integrated by CUPRAC, FRAP, TEAC, thiol, PON-1, TOS, POX-Act, d-ROMs, AOPP and TBARS can be measured in WB and RBCs of the dog. Changes in the redox status assays were observed in WB and RBCs lysates after the addition of ascorbic acid, being analytes in WB more sensitive to detect these changes in our experimental conditions. This higher sensitivity to the detection of changes and its easier sample preparation makes WB a promising sample for the evaluation of redox status in dogs. 

## Data Availability

The data supporting the conclusions of this article are included within the manuscript.

## Supplementary Materials

The following are available online at https://www.mdpi.com/article/10.3390/antiox11020424/s1. Figure S1. Linear regression of the antioxidant (a) and oxidant (b) biomarkers validated in whole blood (WB). Regression line showing two WB samples at various dilutions. Regression equation and coefficient of determination (*r*^2^) are shown. Table S1. Mean, standard deviation (SD) and intra- and inter-assay coefficients of variation (CVs, given in %) for antioxidant and oxidant biomarkers obtained in the precision study of assays for validation in two whole blood (WB) samples from dogs (A = Low concentrations, B = High concentrations). Figure S2. Linear regression of the antioxidant (a) and oxidant (b) biomarkers validated in red blood cells (RBCs) lysates. Regression line showing two WB samples at various dilutions. Regression equation and coefficient of determination (*r*^2^) are shown. Table S2. Mean, standard deviation (SD) and intra- and inter-assay coefficients of variation (CVs, given in %) for antioxidant and oxidant biomarkers obtained in the precision study of assays for validation in two red blood cells (RBCs) lysate samples from dogs (A = Low concentrations, B = High concentrations).
