# Supplementary material for: Measurement of Redox Biomarkers in the Whole Blood and Red Blood Cell Lysates of Dogs
Source: Antioxidants (Basel). 2022 Feb 19;11(2):424. doi: 10.3390/antiox11020424 (PMC8869394; doi:10.3390/antiox11020424)
Supplement: Supplementary file 1 [file antioxidants-11-00424-s001.zip › antioxidants-1576930-supplementary.pdf]

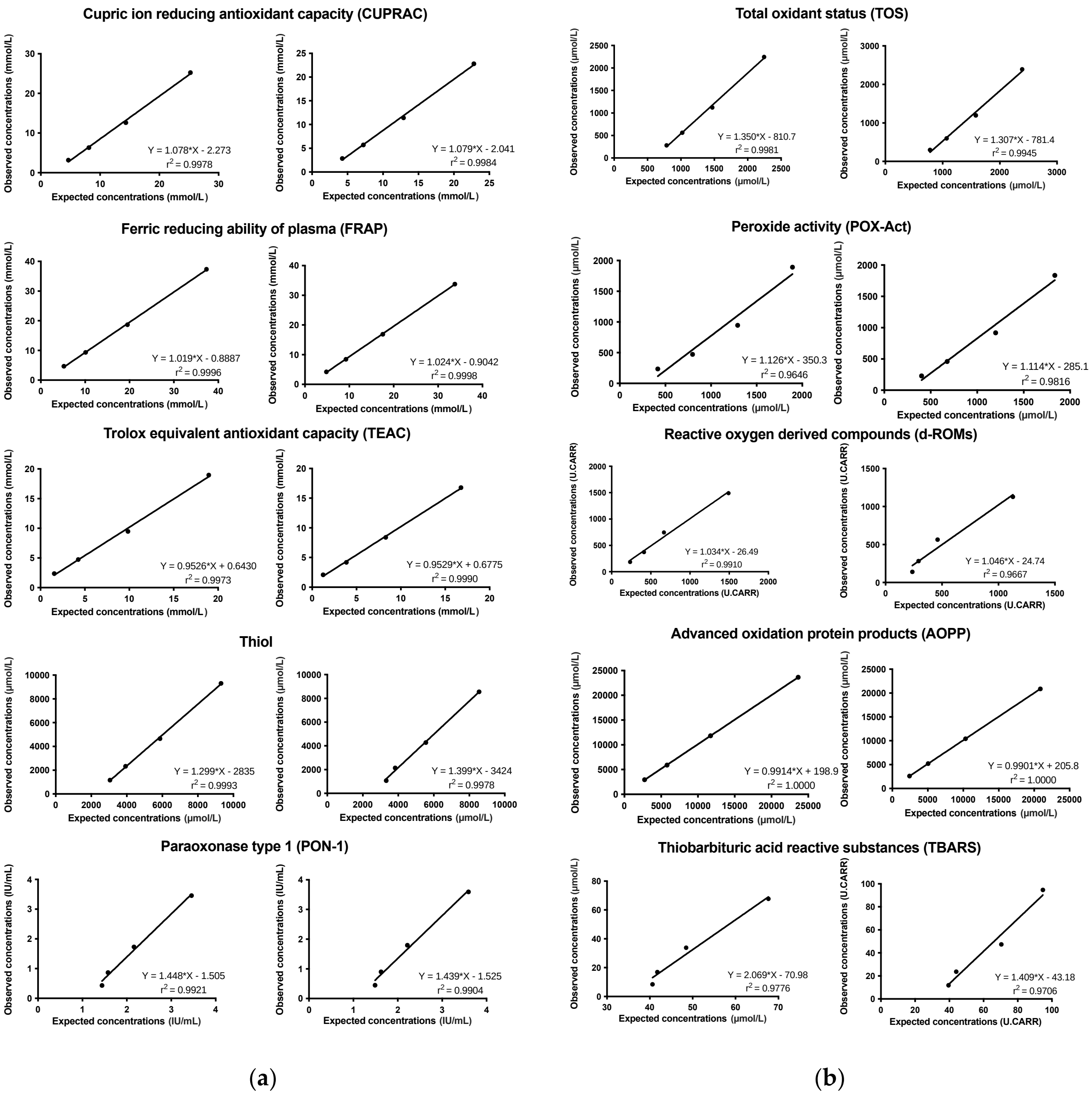

Figure S1. Linear regression of the antioxidant (a) and oxidant (b) biomarkers validated in whole blood (WB). Regression line showing two WB samples at various dilutions. Regression equation and coefficient of determination (r2) are shown.

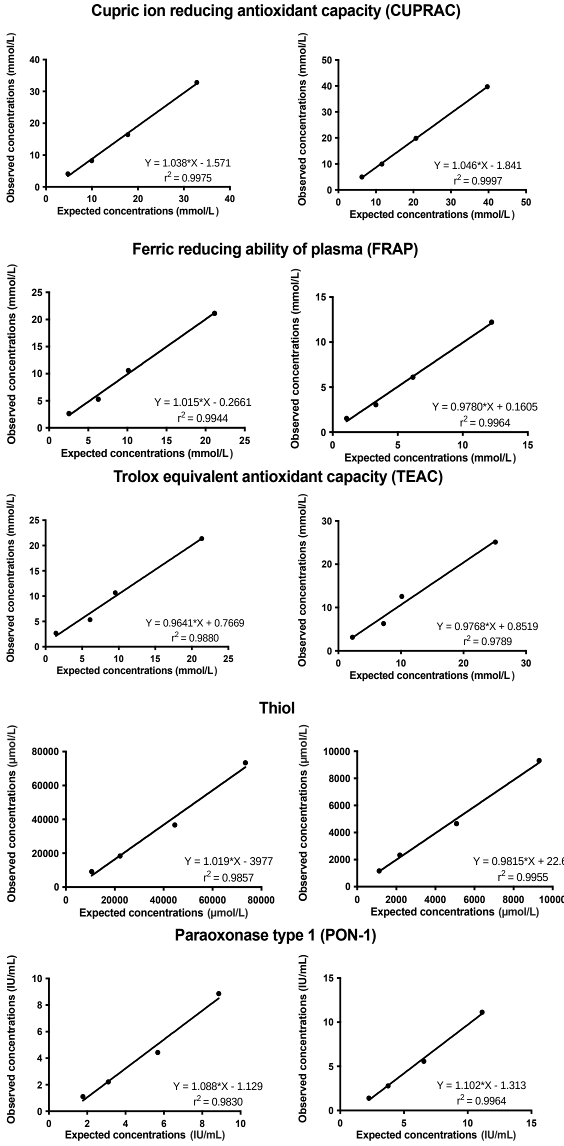

(**a**)

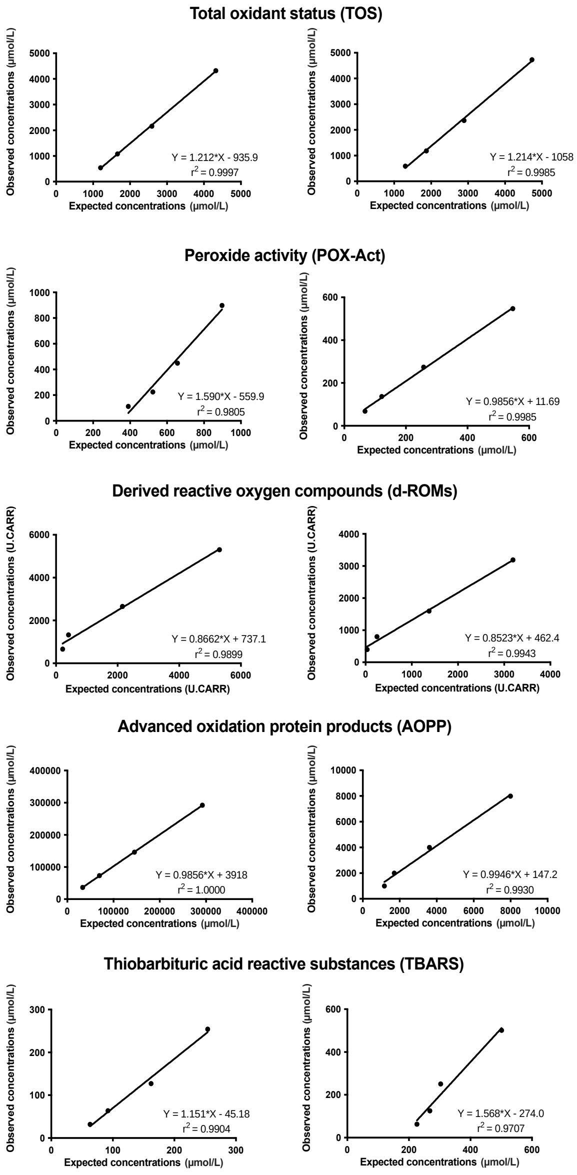

(**b**)

Figure S2. Linear regression of the antioxidant (a) and oxidant (b) biomarkers validated in red blood cells (RBCs) lysates. Regression line showing two WB samples at various dilutions. Re-gression equation and coefficient of determination (r2) are shown.

Table S1. Mean, standard deviation (SD) and intra- and inter-assay coefficients of variation (CVs, given in %) for antioxidant and oxidant biomarkers obtained in the precision study of assays for validation in two whole blood (WB) samples from dogs (A = Low concentrations, B = High concentrations).

		Intra-assay			Inter-assay		
		**Mean**	**SD**	**CV (%)**	**Mean**	**SD**	**CV (%)**
CUPRAC	A	25.81	0.198	0.77	25.53	0.360	0.014
(mmol/L)	B	36.48	0.221	0.61	36.55	0.212	0.06
FRAP	A	20.40	0.260	1.27	20.74	0.593	2.86
(mmol/L)	B	44.31	0.108	0.24	45.25	0.656	1.45
TEAC	A	11.48	0.250	2.14	12.54	0.829	6.61
(mmol/L)	B	22.28	0.242	1.08	23.49	1.425	6.06
Thiol	A	9282	234	0.025	9546	299.612	0.031
(µmol/L)	B	12668	156	0.01	13226	304	0.02
PON-1	A	4.66	0.117	1.88	3.15	0.181	2.86
(IU/mL)	B	6.21	0.056	1.21	5.40	0.154	5.76
TOS	A	2878	40	1.40	2841	30	1.07
(µmol/L)	B	3908	201	5.16	3995	50	1.25
POX-Act	A	527	31	6.04	525	48	9.21
(µmol/L)	B	789	74	9.47	796	55	6.98
d-ROMs	A	226	5.851	2.58	225	10.889	4.82
(U.CARR)	B	411	13.181	3.21	420	12.767	3.04
AOPP	A	24759	92	0.37	25774	622	2.41
(µmol/L)	B	36152	110	0.31	37313	882	2.36
TBARS	A	46.33	7	15.89	39.74	5.414	13.62
(µmol/L)	B	64.76	9	15.41	67.01	4.946	7.38

Table S2. Mean, standard de-viation (SD) and intra- and inter-assay coefficients of variation (CVs, given in %) for antioxidant and oxidant biomarkers obtained in the precision study of assays for validation in two red blood cells (RBCs) lysate samples from dogs (A = Low concentrations, B = High concentrations).

		**Intra-assay**			**Inter-assay**		
		**Mean**	**SD**	**CV (%)**	**Mean**	**SD**	**CV (%)**
CUPRAC (mmol/L)	A	28.28	0.64	2.27	27.55	0.245	0.89
	B	35.31	0.91	2.60	35.35	0.204	0.57
FRAP (mmol/L)	A	34.91	0.656	1.88	34.14	0.653	1.91
	B	47.05	0.503	1.07	46.80	1.267	2.71
TEAC (mmol/L)	A	17.72	0.279	1.57	18.74	1.713	9.14
	B	23.15	0.436	1.88	22.55	0.850	3.768
Thiol (μmol/L)	A	7824	281	3.59	9034	675	7.48
	B	10036	234	2.34	10414	406	4.64
PON-1 (IU/mL)	A	0.54	0.025	2.24	0.72	0.120	8.09
	B	1.12	0.022	3.96	1.33	0.058	8.96
TOS (μmol/L)	A	2998	34	1.17	2962	24	0.84
	B	4008	93	2.33	4044	52	1.30
POX-Act (μmol/L)	A	175	11	6.66	165	15	9.58
	B	521	3	0.74	498	24	4.97
d-ROMs (U.CARR)	A	370	154	4.53	377	26	15.98
	B	1431	17	10.79	1630	260	7.13
AOPP (μmol/L)	A	24522	250	1.02	21684	533	2.46
	B	33218	351	1.06	30020	853	2.84
TBARS (μmol/L)	A	127	5	4.07	125	11	9.41
	B	211	21	9.84	176	22	12.60
